# Prevention of Type 2 Diabetes by Lifestyle Changes: A Systematic Review and Meta-Analysis

**DOI:** 10.3390/nu11112611

**Published:** 2019-11-01

**Authors:** Matti Uusitupa, Tauseef A. Khan, Effie Viguiliouk, Hana Kahleova, Angela A Rivellese, Kjeld Hermansen, Andreas Pfeiffer, Anastasia Thanopoulou, Jordi Salas-Salvadó, Ursula Schwab, John L. Sievenpiper

**Affiliations:** 1 Institute of Public Health and Clinical Nutrition, School of Medicine, University of Eastern Finland, P.O. Box 1627, 70211 Kuopio, Finland; ursula.schwab@uef.fi; 2 Toronto 3D Knowledge Synthesis and Clinical Trials Unit, Clinical Nutrition and Risk Factor Modification Centre, St. Michael’s hospital, Toronto, ON M5B 1W8, Canada; tauseef.khan@utoronto.ca (T.A.K.); effie.viguiliouk@utoronto.ca (E.V.); john.sievenpiper@utoronto.ca (J.L.S.); 3 Department of Nutritional Sciences, Faculty of Medicine, University of Toronto, Toronto, ON M5S 1A1, Canada; 4 Physicians Committee for Responsible Medicine, Washington, DC 20016, USA; hana.kahleova@gmail.com; 5 Institute for Clinical and Experimental Medicine, 140 21 Prague, Czech Republic; 6 Department of Clinical Medicine and Surgery, Federico II University, 80138 Naples, Italy; rivelles@unita.it; 7 Department of Endocrinology and Internal Medicine, Aarhus University Hospital, 8200 Aarhus N, Denmark; kjeld.hermansen@clin.au.dk; 8 German Institute of Human Nutrition Potsdam-Rehbrücke, Clinical Nutrition-DZD, Arthur-Scheunert-Allee 114-116, D-14558 Nuthetal, Germany; andreas.pfeiffer@charite.de; 9 Department of Endocrinology, Charité University Medicine, Diabetes and Nutrition, Campus Benjamin Franklin, Hindenburgdamm 30, D-12203 Berlin, Germany; 10 German Center for Diabetes Research (DZD), 85764 München-Neuherberg, Germany; 11 Diabetes Center, 2nd Department of Internal Medicine, Medical School, National and Kapodistrian University of Athens, Hippokration General Hospital of Athens, 157 72 Athens, Greece; a_thanopoulou@hotmail.com; 12 Human Nutrition Unit, University Hospital of Sant Joan de Reus, Department of Biochemistry and Biotechnology, Faculty of Medicine and Health Sciences, Institut d’Investigació Sanitària Pere Virgili, Rovira i Virgili University, 43201 Reus, Spain; jordi.salas@urv.cat; 13 Centro de Investigación Biomédica en Red Fisiopatología de la Obesidad y la Nutrición (CIBEROBN), Institute of Health Carlos III, 28029 Madrid, Spain; 14 Department of Medicine, Endocrinology and Clinical Nutrition, Kuopio University Hospital, 70210 Kuopio, Finland; 15 Division of Endocrinology and Metabolism, Department of Medicine, St. Michael’s Hospital, Toronto, M5B 1W8 ON, Canada; 16 Li Ka Shing Knowledge Institute, St. Michael’s Hospital, Toronto, M5B 1T8 ON, Canada

**Keywords:** prevention, type 2 diabetes, diet, lifestyles, complications

## Abstract

Prevention of type 2 diabetes (T2D) is a great challenge worldwide. The aim of this evidence synthesis was to summarize the available evidence in order to update the European Association for the Study of Diabetes (EASD) clinical practice guidelines for nutrition therapy. We conducted a systematic review and, where appropriate, meta-analyses of randomized controlled trials (RCTs) carried out in people with impaired glucose tolerance (IGT) (six studies) or dysmetabolism (one study) to answer the following questions: What is the evidence that T2D is preventable by lifestyle changes? What is the optimal diet (with a particular focus on diet quality) for prevention, and does the prevention of T2D result in a lower risk of late complications of T2D? The Grading of Recommendations Assessment, Development, and Evaluation (GRADE) approach was applied to assess the certainty of the trial evidence. Altogether seven RCTs (N = 4090) fulfilled the eligibility criteria and were included in the meta-analysis. The diagnosis of incident diabetes was based on an oral glucose tolerance test (OGTT). The overall risk reduction of T2D by the lifestyle interventions was 0.53 (95% CI 0.41; 0.67). Most of the trials aimed to reduce weight, increase physical activity, and apply a diet relatively low in saturated fat and high in fiber. The PREDIMED trial that did not meet eligibility criteria for inclusion in the meta-analysis was used in the final assessment of diet quality. We conclude that T2D is preventable by changing lifestyle and the risk reduction is sustained for many years after the active intervention (high certainty of evidence). Healthy dietary changes based on the current recommendations and the Mediterranean dietary pattern can be recommended for the long-term prevention of diabetes. There is limited or insufficient data to show that prevention of T2D by lifestyle changes results in a lower risk of cardiovascular and microvascular complications.

## 1. Introduction

Both the prevalence and incidence of type 2 diabetes (T2D) are increasing rapidly worldwide. Worldwide, in 2017, approximately 425 million people had diabetes. This figure may rise to 629 million by 2045. However, the figures for different European countries are not as dramatic as the figures in America and in many low- and middle-income countries. In Europe, the prevalence of T2D is also increasing in parallel to the obesity epidemic. In 2017, the number of patients with diabetes in Europe was 66 million (prevalence 9.1%) and it is estimated to be 81 million by 2045. [1,2]. T2D is a potent risk factor for cardiovascular diseases, but also for blindness, renal failure, and lower limb amputation, decreasing the quality of life of people affected. The burden of diabetes is not only a public health issue, but it also has marked economic consequences. More specifically, the expenses for the treatment of diabetes are increasing mostly due to its long-term complications but also modern drug treatment options [3]. Furthermore, bariatric surgery is becoming more popular for markedly obese patients with T2D due to its significant beneficial effects on metabolic control, long-term complications, and prognosis of T2D [4,5].

The interest in preventing diabetes through lifestyle changes was already present in the 1980s [6], and the opportunity to prevent T2D through lifestyle changes was re-emphasized in the 2004 recommendations of the Diabetes and Nutrition Study Group (DNSG) of the European Association for the Study of Diabetes (EASD) [7]. Since then, a number of randomized controlled trials (RCTs) have been published that show that T2D is preventable, or its onset can be markedly postponed, by increasing physical activity, reducing weight, and changing dietary habits.

To update the evidence for the EASD clinical practice guidelines for nutrition therapy, we conducted a systematic review and, where appropriate, meta-analyses of the available randomized controlled trials assessing lifestyle interventions in the prevention of T2D with the aim of answering the following questions:

(a) What is the evidence that T2D is preventable by lifestyle changes in adults with impaired glucose tolerance (IGT) and (b) what are the long-term results on the prevention of T2D?
What is the evidence that the lifestyle changes aimed to prevent T2D also modify the risk of cardiovascular disease and microvascular complications in people with IGT?
What is the optimal dietary composition for the prevention of T2D in people with IGT?

A systematic review and meta-analysis of randomized controlled trials (RCTs) was conducted to assess the role of lifestyle changes on the prevention of T2D using the Grading of Recommendations Assessment, Development, and Evaluation (GRADE) approach. In addition, we discuss the lifestyle including dietary changes that have been successfully used for the prevention of T2D and summarize the long-term follow-up results after the active intervention periods from the major T2D prevention trials on the incidence of T2D and micro- and macrovascular diseases, and finally make the conclusions regarding the three study questions.

We attempt to answer these three questions in turn, summarizing the evidence following by making conclusions at the end of the paper.

## 2. Evidence That T2D Is Preventable by Changing Lifestyles

A systematic review and meta-analysis of randomized controlled trials (RCTs) was conducted to assess the role of lifestyle changes on the prevention of T2D using the Grading of Recommendations Assessment, Development, and Evaluation (GRADE) approach.

## 3. Methods

### 3.1. Search Strategy and Study Selection

We conducted our systematic review and meta-analysis according to the Cochrane Handbook for Systematic Reviews of Interventions [8], and reported the results according to the PRISMA guidelines (www.prisma-statement.org). We conducted standard literature searches of PubMed (MEDLINE), EMBASE, and Cochrane Library through 21 June 2019 to identify both original RCTs and recent systematic reviews [9,10,11,12] that have examined the association of lifestyle intervention with T2D. The following key words were used in selecting original RCTs for this search: type 2 diabetes, RCT, prevention, systematic reviews, impaired glucose tolerance (IGT), diet, dietary pattern, physical activity, and lifestyle. We supplemented the systematic search with a manual search of reference lists. We selected RCTs comparing the effect of lifestyle intervention (exercise-plus-diet or exercise-plus-diet-plus-weight loss) versus control (no lifestyle intervention) on incident T2D defined using study-specific criteria based on a 2 h oral glucose tolerance test (OGTT) in all populations in an outpatient setting with a minimum follow-up of 1 year. We included studies that were conducted in a high-risk population including those with IGT and metabolic syndrome. Studies that only assessed exercise intervention without diet or weight-loss, used a drug(s) as part of the lifestyle intervention, or only reported observational cohort studies were excluded. In case of the multiple publication of the same trial, we used the one with the end-trial data.

### 3.2. Data Extraction

Two investigators (EV and TAK) independently reviewed and extracted relevant data from each included report. A standardized form was used to extract data on sample size, participant characteristics, study setting and design, level of monitoring of eating habits, intervention and control arm, macronutrient composition of diets, energy balance, follow-up duration, funding source and outcome data. All discrepancies and disagreements were resolved through consensus.

### 3.3. Risk of Bias Assessment

Included trials were independently assessed by two investigators (EV and TAK) for the risk of bias using the Cochrane Risk of Bias Tool [8]. An assessment was performed across 5 domains of bias (sequence generation, allocation concealment, blinding, incomplete outcome data and selective reporting). The risk of bias was assessed as either low (proper methods taken to reduce bias), high (improper methods creating bias) or unclear (insufficient information provided to determine the bias level). All discrepancies and disagreements were resolved through consensus or, where necessary, by a third author (JLS). The methods applied are described in the individual publications [13,14,15,16,17,18,19,20,21,22,23,24,25,26,27,28].

### 3.4. Data Syntheses

All analyses were conducted using Stata 16 ((StataCorp, College Station, TX, USA). Data were expressed as risk ratios (RRs) with 95% confidence intervals (CIs) and pooled using the restricted maximum likelihood (REML) random-effects models [29]. A random-effects model assumes that study estimates are estimating different, yet related, intervention effects and thus incorporates heterogeneity among studies. This is a more appropriate method to pool studies that may differ slightly in distribution of risk factors, population, size, and outcomes [30]. Heterogeneity was assessed using the Cochran Q statistic and quantified using the I^2^ statistic. Significance for heterogeneity was set at *p* < 0.10, with an I^2^ > 50% considered to be evidence of substantial heterogeneity [15]. Sources of heterogeneity were explored using sensitivity and subgroup analyses. Sensitivity analyses were performed in which each individual trial was removed from the meta-analysis and the effect size recalculated to determine whether a single trial exerted an undue influence. If ≥10 trials were available, then a priori subgroup analyses were conducted using meta-regression by baseline values, study design, follow-up, comparator arm, risk of bias and diabetes duration [16]. If ≥10 trials were available, then we also assessed publication bias by visual inspection of funnel plots and formal testing by the Egger and Begg tests [17].

### 3.5. Grading of the Evidence

The GRADE approach was used to assess the certainty of the evidence [18,19,20,21,22,23,24,25,26,27,28]. The certainty of the evidence was graded as high, moderate, low, or very low. Randomized controlled trials receive an initial grade of high by default and are downgraded based on the following pre-specified criteria: risk of bias (weight of trials showing risk of bias by the Cochrane Risk of Bias Tool), inconsistency (substantial unexplained inter-study heterogeneity, I^2^ > 50% and *p* < 0.10), indirectness (presence of factors that limit the generalizability of the results), imprecision (the 95% CI for effect estimates were wide or cross minimally important differences (MIDs) for benefit or harm), and publication bias (significant evidence of small-study effects). The MID for T2D was set at 5 percent based on increased cardiovascular disease risk [31].

## 4. Results

### 4.1. Search Results

**Figure 1:**
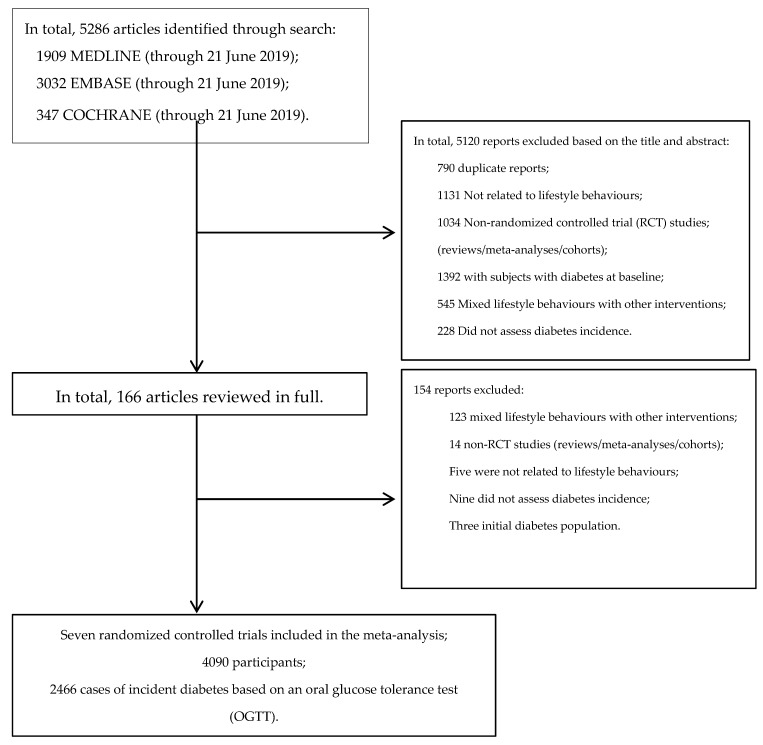
Flow diagram outlining the systematic search and article selection process.

Figure 1 outlines our systematic search. We identified 5286 articles from PubMed (MEDLINE), EMBASE, and Cochrane Library.

### 4.2. Randomized Controlled Trials

We identified seven RCTs comprising 4090 study participants and 2466 incident type 2 diabetes cases [32,33,34,35,36,37,38,39,40] (see Table 1 and Figure 1). Except for the study by Bo et al. [38,39] (which was conducted in people with dysmetabolism), all studies were carried out in people with impaired glucose tolerance (IGT) based on an OGTT, and the diagnosis of incident diabetes was confirmed by OGTT applying contemporary WHO criteria for diabetes mellitus. Detailed data on the intervention measures and the follow-up of the control groups have been reported in individual publications and summarized in Table 1.

### 4.3. Risk of Bias

**Figure 2:**
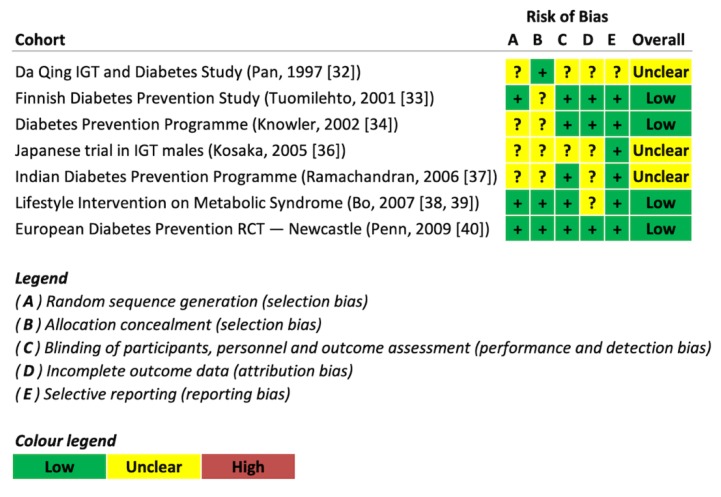
Risk of bias assessment.

Figure 2 shows the individual Cochrane Risk of Bias assessments of seven trials included in the current meta-analysis (see Figure 1 and Table 1 for details). The majority of trials were judged as having unclear or low risk of bias across domains. No evidence of a serious risk of bias was detected.

### 4.4. Effect of Lifestyle Changes on Type 2 Diabetes Risk

**Figure 3:**
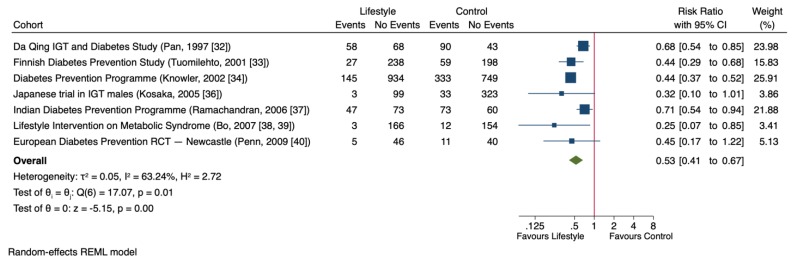
Forest plot of randomized controlled trials investigating the effect of lifestyle changes on type 2 diabetes risk (T2D). The pooled effect estimate for the overall effect is represented by the green diamond. Data are expressed as weighted risk ratios with 95% confidence intervals (CIs) using the restricted maximum likelihood (REML) random-effects model. Inter-study heterogeneity was tested by the Cochrane Q-statistic at a significance level of *p* < 0.10 and quantified by I^2^, where a level of ≥50% represented substantial heterogeneity.

Figure 3 shows the effect of lifestyle changes on T2D risk based on the meta-analysis. In seven trials involving 4090 participants [32,33,34,36,37,38,40], lifestyle intervention significantly decreased T2D risk compared to control groups (RR = 0.53 (95% CI: 0.41, 0.67), *p* < 0.001), with evidence of substantial inter-study heterogeneity (I^2^ = 63%, *p* = 0.01).

### 4.5. Sensitivity and Subgroup Analyses

Table 2 shows selected sensitivity analyses in which the systematic removal of individual trials altered the results. The evidence of substantial heterogeneity was partially explained by the removal of Knowler et al. [34], which changed the evidence for heterogeneity from significant (I^2^ = 65%, *p* = 0.009) to non-significant (I^2^ = 43%, *p* = 0.16). However, this did not appreciably change the overall effect estimate (RR = 0.49 (95% CI: 0.37, 0.64), *p* < 0.001). Subgroup analyses were not conducted for any outcome as <10 trials were available.

### 4.6. Publication Bias

Publication bias was not assessed for any outcome as <10 trials were available.

### 4.7. GRADE Assessment

Table 3 shows a summary of the GRADE assessments of the overall certainty of the effect of lifestyle changes on the risk of transition from IGT to T2D. The evidence was graded as high for the effect of lifestyle intervention on T2D risk reduction without any downgrading for risk of bias, inconsistency, indirectness, imprecision, or other considerations.

## 5. Discussion on the Systematic Review and Meta-Analysis

We conducted a systematic review and meta-analysis of seven randomized controlled trials involving 4090 predominantly middle-aged participants with glucose impairment (IGT or dysmetabolism), which showed that lifestyle modification including improved diet and physical activity reduced the risk of type 2 diabetes by 47 percent.

### 5.1. Results in the Context of Existing Literature

Recent systematic reviews published on the prevention of T2D in high-risk groups uniformly conclude that the onset of T2D can be delayed or prevented with lifestyle changes. Furthermore, these systematic reviews conclude that lifestyle changes may result in the sustained reduction of T2D [9,10,11,12]. On the other hand, a recent Cochrane review concluded that the evidence took into account only the combined effect of physical activity and dietary changes, and the evidence on the effect of diet or physical activity alone is insufficient [12].

A brief discussion of the included studies and other literature is helpful here as these will also be referred to in the subsequent sections of this paper. The Chinese Da Qing study [32] had altogether 577 IGT individuals in 33 study clinics that were randomized to control, exercise, healthy diet, and healthy diet plus exercise clinics, with a follow-up of 6 years. The risk of diabetes was reduced by 33% in the diet-only group, 47% in the exercise-only group and 38% in the diet-plus-exercise group as compared to the control group, without significant differences between the intervention groups. The study individuals were normal weight or overweight at baseline, and the reduction in total energy intake was 100–240 kcal depending on the intervention (Table 1).

In the Finnish Diabetes Prevention Study (FDPS) [33], 522 individuals with IGT were randomized into a control or lifestyle intervention group (healthy diet and physical activity promotion). The diagnosis of T2D was based on repeated OGTT. After 3.2 years of follow-up, there was a significant decrease in the incidence of T2D, and the trial was prematurely stopped based on the decision of the independent advisory committee. The risk reduction was 58% in the intervention group compared to the control group. Weight loss was larger in the intervention group: the difference in weight reduction between the groups was 3.5 and 2.6 kg at 1 and 3 years, respectively. The intervention group also showed an increase in physical activity and the number of sedentary people was smaller in the intervention (17%) than in the control group (29%).

In the Diabetes Prevention Program (DPP) study conducted in the USA [34], altogether, 3234 individuals with IGT in 27 centers were randomized into the lifestyle intervention, metformin or control groups. The mean follow-up was 2.8 years. The risk of T2D was reduced by 58% in the lifestyle intervention group as compared to the control group. In the metformin group, the risk of diabetes was 31% lower than in the control group. At year 1, weight reduction in the intervention group was 5.6 kg and 0.1 kg in the control group. No detailed changes in physical activity were reported. It is of note that the initial BMI in the DPP was 34 kg/m^2^ when in the FDPS it was 30–31 kg/m^2^.

In a Japanese study on 458 men with IGT [36], compared to the control group, a remarkable relative risk reduction of 67.4% was found in the intervention group that aimed for weight reduction, increased vegetable intake and physical activity during the 4 year follow-up. The BMI goal was 22 kg/m^2^ and the majority of participants had either normal BMI or they were overweight with IGT. Still, the average weight loss was 2.2 kg in the intervention group.

In the Indian Diabetes Prevention Programme (IDPP-1) study [37], consisting of 531 subjects with IGT, there was a 28.5% reduction in the risk of T2D after 3 years of follow-up in the lifestyle modification group (LSM) compared to the control group, 28.2% reduction in the LSM-plus-metformin (Met) group and 26.4% reduction in the Met group. No significant group differences were found in the preventative effect with regard to LSM, Met and LSM-plus-Met groups. This study did not report significant changes in body weight.

Bo et al. in Italy carried out a lifestyle intervention aimed at the prevention of metabolic syndrome (MetS) in 335 subjects with dysmetabolism. This group included subjects with metabolic syndrome together with those having only two components of metabolic syndrome plus high hs-CRP values. In addition to an effect on metabolic syndrome, this study also reported 1 and 4 year results on the incidence of T2D [38,39]. After one year, there was a marked risk reduction in the incidence of T2D [OR 0.23; 95% CI 0.06–0.85]. The difference in weight reduction between the intervention and control groups was approximately 2.3 kg. After 4 years, the incidence of T2D was 5.4% in the intervention group and 10.2% in the control group.

In the Newcastle arm of the European Diabetes Prevention Study (EDIPS) study [40] consisting of 102 subjects with IGT, after 3 years of lifestyle intervention following mostly principles of the FDPS, the incidence of T2D was 5.0% and 11.1% in the intervention and the control groups, respectively. The average weight loss was 2.5 kg in the intervention group and sustained beneficial changes in lifestyles predicted better outcome in the T2D risk.

Before the above randomized trials that are included in the meta-analysis, Eriksson and Lindgarde reported in 1991 [41] that a 6 month sequential intervention of dietary change or increased physical activity may have prevented the development of T2D in 181 Swedish men who volunteered to take part in the lifestyle intervention compared to those who did not volunteer to participate.

In a smaller study of 88 subjects (the SLIM Study) [35], with 2 years of lifestyle intervention, not included in the current meta-analysis because it did not fulfill the inclusion criteria, there was a significant improvement in 2 h glucose values in the active intervention group. The beneficial changes could be ascribed to moderate weight loss and dietary changes (i.e., reduction in saturated fat intake) in combination with increased physical activity. Incidence data on T2D after 3 years were included in the European Diabetes Prevention Study RCT [42], where the preventative effect of ≥5% weight loss was particularly high, especially if maintained for 3 years.

Two post-hoc reports from the PREDIMED study also suggest that it is possible to prevent T2D even without significant weight loss in individuals at high risk for cardiovascular disease (CVD), using the Mediterranean diet including extra virgin olive oil or nuts. The risk reduction using the Mediterranean diet intervention, either supplemented with virgin olive oil or nuts, compared to the control group was 30% to 50% depending on the baseline population [43,44]. These studies are discussed in greater detail later in the manuscript with regard to the optimal diet for the prevention of T2D and cardiovascular disease.

### 5.2. Strengths and Limitations

Our systematic review and meta-analysis have several strengths. These include a rigorous search and selection strategy that identified all available randomized controlled trials examining the effect of lifestyle modification on T2D in individuals; the inclusion of predominantly high-quality randomized controlled trials, which give the greatest protection against bias; the use of the REML random-effects model, which is robust to non-normal distributions and has been recommended for use in meta-analyses over other random-effects estimators [29]; and the assessment of the overall certainty of the evidence using the GRADE approach.

There were no major limitations of our systematic review and meta-analysis. There was an issue of high heterogeneity, but we did not downgrade for the observed inconsistency. We did not consider the statistical heterogeneity to be a limitation as our meta-analysis included large studies with narrow confidence intervals and similar estimates in the same direction. Therefore, this apparent inconsistency was an artefact of non-overlapping narrow CIs rather than a limitation of the certainty of the overall estimate [23,45]. Balancing the strengths and limitations, the evidence as assessed using GRADE was of high certainty for the effect of lifestyle modification on the reduction of T2D.

## 6. Long-Term Results on the Prevention of Type 2 Diabetes

Three follow-up studies, the Da Qing Chinese study [46], FDPS [47,48] and DPP [49], showed that the beneficial lifestyle changes achieved in the prevention of T2D trials resulted in a sustained risk reduction of T2D over 10 years of follow-up (Table 4).

**Figure 4:**
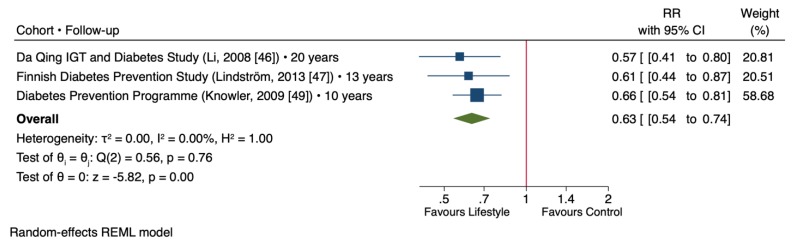
Forest plot of randomized controlled trials investigating the long-term post-intervention effect of lifestyle changes on type 2 diabetes risk. The pooled effect estimate for the overall effect is represented by the green diamond. Data are expressed as weighted risk ratios with 95% confidence intervals (CIs) using the REML random-effects model. Inter-study heterogeneity was tested by the Cochrane Q-statistic at a significance level of *p* < 0.10 and quantified by I^2^, where a level of ≥50% represented substantial heterogeneity.

Figure 4 shows the effect of lifestyle changes on the T2D risk based on the meta-analysis of the selected trials that had the long-term follow-up after the lifestyle intervention phase. In three trials consisting a total of 3855 participants with a median follow-up of 13 years [46,47,49], lifestyle intervention was associated with significantly lower T2D risk compared to control groups (RR = 0.63 [95% CI: 0.54, 0.74], *p* < 0.001) with no evidence of inter-study heterogeneity (I^2^ = 0%, *p* = 0.76).

Based on the results from FDPS [47,48], 22 subjects with IGT must be treated for one year or 5 subjects for five years to prevent one case of diabetes. Accordingly, in DPP [49], the respective figure was 6.9 subjects for a 3 year intervention.

## 7. Evidence That the Prevention of T2D in High-Risk Individuals Results in a Lower Risk of Cardiovascular Disease (CVD) and Microvascular Complications

Among the selected intervention trials, three follow-up post-intervention studies reported cardiovascular and/or microvascular complications (Table 5). Furthermore, we considered the PREDIMED intervention trial results for this question as this study was carried out in high-risk individuals [43,44].

This question is of particular importance, since the ultimate goal of the prevention and treatment of diabetes is the prevention of the long-term complications of diabetes associated with long-term hyperglycemia, dyslipidemias, hypertension, and other metabolic abnormalities, including low-grade inflammation [50]. Indeed, long-term intervention trials on the prevention of T2D have shown that besides improved glycemia, due to the correction of insulin resistance and possibly the preservation of beta-cell capacity [33,34,51], many of the well-known cardiovascular risk factors and characteristics of metabolic syndrome are corrected by changing to a healthier diet, increasing physical activity and losing weight [43,44,51,52,53]. However, there has been little evidence that the incidence of CVD or microvascular complications can be postponed or prevented by changing lifestyles. Recent data from the Da Qing Diabetes Prevention Outcome study reported results for both mortality and morbidity that suggest long-term benefits as a result of changing lifestyle habits. To summarize, there was a significant reduction in all cause deaths (26%), CVD deaths (33%) and total CVD events (26%) in the combined intervention groups as compared to the control group. Furthermore, composite microvascular diseases (35%) and the incidence of any retinopathy (40%) were significantly lower in the combined intervention groups in this cohort [54].

Furthermore, the PREDIMED study reported a significant reduction in combined stroke and all cardiovascular events in individuals randomized to the Mediterranean diet (MedDiet) plus extra-virgin olive oil or MedDiet plus nuts group [58]. Recently, the incidence of retinopathy was reported to be lower in the PREDIMED study in individuals randomized to MedDiet plus extra-virgin olive oil group (RR 0.56; 95%CI 0.32–0.97) or MedDiet plus nuts group (0.63; 95% CI 0.35–1.11). By contrast, no effect of the Mediterranean diet interventions on diabetic nephropathy was reported in the PREDIMED [59]. In the DPP follow-up study [55], retinopathic changes in women were lower in the former lifestyle intervention group than in the control group. Similarly, individuals who developed T2D had higher incidence of retinopathy than those who were non-diabetic after a long follow-up period (Table 5). In FDPS, no difference was found in CVD morbidity or mortality between the intervention and control groups after 10 years, but incident cases remained low in both intervention and control groups [56]. In a sub-group analysis, the occurrence of retinopathy (microaneurysms) was significantly higher in the control (37/98, 38%) than in the intervention group (27/113, 24%; *p* = 0.026, see Table 4 for adjusted results) of the former FDPS participants [56].

An original report from the Look AHEAD trial showed no benefit of lifestyle intervention for the prevention of cardiovascular disease in patients with T2D, but a post-hoc analysis showed a 21% risk reduction in combined cardiovascular events in individuals who were able lose at least 10 kg of body weight as compared to patients with a stable body weight or long-term weight gain [60].

A recent systematic review and meta-analysis of prospective cohort studies and randomized clinical trials suggests that MedDiet has a beneficial role on the CVD prevention in populations inclusive of the individuals with T2D [61].

### Discussion on Macro- and Microvascular Risk Reduction in the T2D Prevention Trials

Among the diabetes prevention trials which have examined follow-up data, only the Chinese Da Qing Diabetes Prevention Outcome Study has reported lower mortality and morbidity from any cause and cardiovascular disease in the people with IGT randomized into lifestyle intervention groups (Table 5). Furthermore, the Chinese study found a clear decrease in composite microvascular diseases and retinopathy [54]. Indeed, these long-term results are of particular interest, since one long-term goal of the prevention of T2D is to prevent its complications as well. A longer follow-up of a relatively younger age cohort that is also less obese is a possible reason why significant risk reduction in CVD mortality and morbidity is only seen in the Chinese study and not in the American DPP Outcome Study [55] or in the FDPS [56]. After the active intervention phase, both the American and Finnish study participants, on average, remained relatively obese compared to the Chinese study. There may also be genetic or ethnic differences between the study populations, resulting in different distributions of the risk factors for T2D and of T2D rate itself [3]. For example, smoking was particularly common among the Chinese study participants [54]. Furthermore, the management of the main risk factors and health care resources available may offer other explanations for divergent results. In terms of microvascular complications, which are closely associated to hyperglycemia, the Chinese study results were encouraging with a 35% reduction in composite microvascular complications and 40% reduction in any retinopathy in the intervention groups. The results from both the DPP Outcome Study and the FDPS supported the long-term benefit achieved by changing lifestyles with regard to incident retinopathy [55,57]. Finally, it should be emphasized that the statistical power of the intervention studies on the prevention of T2D may not be sufficient to show significant differences in CVD outcomes between the intervention and the control groups [62].

## 8. Discussion on the Factors Explaining the Risk Reduction of T2D Including the Optimal Dietary Composition for the Prevention of T2D

### 8.1. What Are the Factors Explaining the Risk Reduction of T2D in Randomized Controlled Trials?

This question is of particular importance as it is related to strategies in preventing T2D. The Da Qing IGT study is the only study with both diet and physical activity arms randomized by clinic [32], and the PREDIMED trial is the only study testing the effect of a food pattern enriched with key foods (nuts or virgin olive oil) without physical activity or energy restriction [43,44]. All other lifestyle intervention studies combine dietary changes, weight reduction for overweight or obese people, and physical activity. It is of note that Chinese people with IGT in the Da Qing study [32], Japanese men with IGT [36], and individuals in the Indian IDD-1 study [37] had a much lower BMI than in study populations carried out in Europe or in the U.S.A.

### 8.2. Weight Reduction

Based on secondary analyses of randomized controlled trials, it can be concluded that a better adherence to lifestyle changes in general results in the better long-term prevention of T2D [33,48,49]. Furthermore, based on the evidence coming from observational studies on T2D risk factors [2,63] and the remarkable beneficial effects of weight reduction on glucose metabolism [51,64,65,66], weight reduction has been considered as a cornerstone in the prevention of T2D; with larger weight reductions associated with a lower risk of T2D. In the EDIPS study on 771 participants with IGT combining data from the FDPS, and SLIM and Newcastle studies, the risk of T2D was 89% lower in individuals who were able to sustain weight loss of at least 5% over 3 years than in individuals without significant weight changes [42]. Nevertheless, it is impossible to conclude that weight reduction is the only means to reduce the risk of T2D in overweight and obese people with impaired glucose metabolism, since weight loss is almost always associated with simultaneous changes in physical activity and/or diet. Indeed, the studies in people with Asian origin suggest that changing diet and increasing physical activity also seem to play a significant role in the prevention of T2D in individuals at risk for T2D with both normal body weight and over-weight people [32,36,37]. The importance of weight reduction in T2D can be gauged from a recent weight-management trial, in which 306 individuals with T2D in 39 primary care practices demonstrated a remission rate of 86% in individuals who lost 15 kg or more (24% of participants) [67]; an overall weight-loss difference of 9 kg resulted in a remission rate of 46% in the intervention group versus 4% in the control group in the full study.

### 8.3. Optimal Diet

#### 8.3.1. Individual Nutrients and Foods

Several observational studies have been conducted to analyze the associations between food groups or nutrient consumption and T2D incidence. Ley et al. [68] conducted a series of meta-analyses of prospective cohort studies on food and beverage intake and T2D risk. Processed and unprocessed red meat, white rice, and sugar-sweetened beverages have shown a consistent positive relation with T2D, whereas green leafy vegetables, total dairy products, whole grains, alcohol in moderation in women, and coffee have been inversely associated with T2D. The consumption of berries and fruits rich in anthocyanins, such as bilberries, blueberries, grapes, apples, and pears, has also been associated with a lower risk of T2D [69]. Recent evidence also shows that yogurt intake [70] and nut intake (in women) is inversely associated with T2D. Legumes are another food group with cardiometabolic benefits [71,72,73,74,75,76,77] and legumes show an inverse association with the risk of diabetes and gestational diabetes [77,78]. In the same meta-analysis of prospective studies by Ley et al. [68], heme-iron, glycemic index and glycemic load of the diet were directly associated with T2D incidence, whereas total magnesium and vitamin D in the diet, as well as cereal fiber, were inversely related to T2D. A recent review based on meta-analyses and earlier reviews emphasize the preventive effect of whole grains and dietary fiber on the incidence of T2D [79].

#### 8.3.2. Dietary Patterns

In addition to individual nutrients and foods, several studies have looked at dietary patterns and prevention of T2D. A Western dietary pattern, which is high in sugar-sweetened soft drinks, refined grains, diet soft drinks, and processed meat, was associated with an increased risk of diabetes in the Nurses Health Study (NHS) I and NHS II studies [80].

In contrast, some prospective cohort studies have demonstrated that adherence to plant-based dietary patterns, such as Mediterranean [81,82] DASH (Dietary Approaches to Stop Hypertension) or vegetarian dietary patterns [82,83,84,85], are associated with a lower risk of T2D incidence. In two prospective studies, a Mediterranean-type or healthy dietary pattern has also been inversely related to gestational diabetes [78,86].

Meal frequency and timing may also have a role in the T2D risk. Skipping breakfast and snacking have been associated with increased risk of T2D in both men and women [87,88]. Based on limited evidence, consuming breakfast regularly and not eating snacks between main meals may also be a strategy to reduce the risk of T2D [89].

#### 8.3.3. Diet and Weight Loss

Current evidence from randomized intervention trials (Table 1) suggests that weight loss by means of a healthy diet with lower saturated fat intake, but rich in vegetables, fruit, and whole grain products is beneficial in the prevention of T2D, especially when combined with physical activity. Indeed, all of the seven randomized lifestyle intervention studies in our systematic review and meta-analysis applied this kind of dietary approach. In FDPS, the best results in the prevention of T2D were achieved in IGT individuals with high fiber but moderate fat intake [47,90]. Similarly, in the American DPP study, 1 year weight loss success was associated with a high carbohydrate, high fiber, but a rather low total and saturated fat diet intake [91]. Regarding the quality of dietary fat, current evidence suggests that unsaturated fatty acids may have beneficial effects on insulin sensitivity and it is suggested to lower the risk of T2D [92,93].

In the PREDIMED trial, the Mediterranean diet enriched in nuts or extra virgin olive oil, resulted in a significant reduction in the incidence of T2D independent of weight loss or physical activity changes. This suggests that the quality of the diet may play a role in the prevention of T2D independent of weight changes [43,44]. However, these results are based on post-hoc analyses of a population at high cardiovascular risk and may not be extrapolated to healthy populations. In the SLIM and Newcastle studies, better adherence to the diet also predicted lower T2D risk [42]. To conclude, a diet with low consumption of red and processed meat, sugar, and sugar-sweetened beverages, but rich in vegetables, fruit, legumes, and whole grain products seems to be beneficial in the prevention of T2D.

#### 8.3.4. Physical Activity

The Chinese Da Qing study [32] is the only intervention study that has examined the effect of exercise without weight loss or dietary changes. In the physical activity clinics, the risk of T2D was reduced by 47% as compared to clinics serving as control clinics, but no significant differences were observed between different randomization groups (Table 1). There are no other long-term controlled intervention trials in this field. In FDPS, the impact of physical activity was examined as a secondary analysis taking into account the effect of diet and weight reduction. Based on different criteria used to evaluate physical activity, it was concluded that being physically active may reduce T2D risk by approximately 50% [94]. The recommendations to increase physical activity are strongly grounded by short-term controlled interventions that show improved glucose metabolism after increasing physical activity. Furthermore, epidemiological and trial evidence support the view that physical inactivity/sedentary lifestyle, along with being overweight and/or obese, are important risk factors for T2D and contribute to the current epidemic of T2D [1,2,95,96]. A recent PREDIMED-Plus Trial on overweight/obese individuals with metabolic syndrome who combined an energy-reduced Mediterranean-type diet and exercise promotion showed significant weight reduction (3.2 vs. 0.7 kg) and improvements in glucose metabolism, serum concentrations of triglycerides, HDL-cholesterol, and some inflammatory factors, compared to controls. These results confirm that a multifactorial approach, including physical activity, is successful in the prevention and treatment of disturbances in glucose metabolism [52].

## 9. Conclusions

1. We have a high certainty of evidence that T2D is preventable by changing lifestyle, i.e., weight reduction by diet change according to the current recommendations in terms of quality of fat, fiber intake, increased use of whole grain products, fruit, and vegetables, and increasing physical activity. The risk reduction of T2D is strongly related to the degree of long-term weight loss and adherence to lifestyle changes, and this preventive effect has been demonstrated to sustain for many years after active intervention.
2. Additional well-controlled intervention studies are needed to identify the optimal diet to prevent T2D. Currently, a diet moderate in fat, low in saturated fat intake, rich in fiber, whole grains, and fruit and vegetables, as well as a Mediterranean-type diet, may be recommended for the prevention of T2D in prediabetes.
3. There is still limited/insufficient evidence that the prevention of T2D by changing lifestyle may also prevent CVD or microvascular diseases.

## Figures and Tables

**Table 1:** Summary results on the randomized controlled trials aimed to prevent type 2 diabetes in people with impaired glucose tolerance or in people at high increased risk for diabetes.

Study	Country	N, Characteristics	Study Duration	Risk Reduction of T2D with Lifestyle versus Control	Dietary Goals	Changes in Diet When Available	Physical Activity, Goals/Changes	Comment
Da Qing IGT and Diabetes Study, Pan XR et al. Diabetes Care 1997 [32]	China	In total, 577; all had IGT; 33 health care clinics	6 yrs	Diet 33%; exercise 47%; diet + exercise 38%	Weight reduction in overweight; calorie restriction	CHO 58–60 E%; protein 11 E%; fat 25–27 E%; total calories decrease 100–240 kcal	Increase, e.g., walking	Randomization by clinic; follow-up data available
FDPS, Tuomilehto J et al. N Engl J Med 2001 [33]	Finland	In total, 522; IGT; five centers	3.2 yrs; median 4 yrs	In total, 58%, weight loss; difference 3.5 and 2.6 kg after 1 and 3 yrs, respectively.	Weight reduction >5%; reduce total and SFA; increase dietary fiber	3 yr results: energy reduction 204 kcal; CHO increase 3 E%; fat reduction 5 E%; SFA reduction 3 E%; fiber increase 2 g/1000 kcal	4 h/wk, sedentary people at yr 3: 17% vs. 29% for intervention and control groups, respectively	Individual dietary data and long-term follow-up data available
DPP, Knowler WC et al. New Engl J Med 2002 [34]	USA	In total, 3234; IGT; 27 centers	2.8 yrs	Lifestyle 58%; Metformin 31%; weight loss at yr 1: −5.6 vs. −0.1 kg for intervention vs. control, respectively.	NCEP Step 1; weight loss goal 7%	Energy intake reduction 450 vs. 249 kcal and fat intake reduction 6.6 vs. 0.8 E% for intervention and control, respectively.	150 min/wk	Follow-up data available
Japanese trial in IGT males, Kosaka K et al. Diabetes Res Clin Pract 2005 [36]	Japan	In total, 458 IGT; 356 in control, 102 in intervention, OGTT (100 g glucose dose)	4 yrs	Incidence of T2D 3.0% vs. 9.3%; risk reduction 67.4%; weight loss −2.18 kg	BMI goal 22 kg/m^2^; increase vegetables; reduce food intake by 10%; fat < 50 g/d; alcohol restriction	Not reported	30–40 min walking/d	Normal and overweight men
IDPP-1, Ramachandran A et al. Diabetologia 2006 [37]	India	In total, 531; IGT; lifestyle 133; metformin 133; lifestyle-plus-metformin 129; control 136	30 months	Lifestyle 28.5%; Metformin 26.4%; lifestyle-plus-Metformin 28.2%; no change in body weight	Reduce total calories, refined CHO, fat and sugar; increase high fiber-rich foods	Dietary adherence increased in Intervention groups	Walking 30 min a day	
Lifestyle intervention on metabolic syndrome. Bo S, J Gen Intern Med 2007 [38], Bo S et al. Am J Clin Nutr 2009 [39]	Italy	In total, 375 with dysmetabolism; 169 intervention; 166 control; focus on metabolic syndrome	1 yr,	Risk reduction for T2D 77%, (OR 0.23; 95% CI 0.06–0.85) at year 1.	General recommendations for lose weight and decrease SFA and increase PUFA and fiber	Body weight minus 0.75 vs. plus 1.63 kg; total calories minus 74.6 vs. 43.7 kcal; fat minus 2.64 E%; SFA minus 1.97 E%; CHO 2.14 E%; prot 1.7 E%; NS for control	Increase	4 yrs diabetes incidence 5.4% vs. 10.2% in intervention and control groups, respectively
EDIPS-Newcastle, Penn L. BMC Public Health 2009 [40]	UK	In total, 102; IGT; 51 in intervention and control, respectively	3 yrs	Diabetes incidence 5% vs. 11, 1% yr. body weight change −2.5 kg	Like in FDPS, decrease fat and SFA; increase fiber; body weight reduction	Not reported	Like in FDPS	Sustained beneficial changes in lifestyles predicted better outcome

IGT = impaired glucose tolerance based on OGTT, CHO = carbohydrates, prot = protein, SFA = saturated fatty acids, PUFA = polyunsaturated fatty acids, intervention = intervention group, control = control group, minus = reduction from baseline, NA = not available, and NS = not significant, LSM = lifestyle modification, Met = Metformin. Da Qing IGT: The Da Qing IGT and Diabetes Study; FDPS: Finnish Diabetes Prevention Study; DPP: The Diabetes Prevention Program; IDDP-1: The Indian Diabetes Prevention Programme; EDIPS: European Diabetes Prevention Study; LSM: lifestyle modification; Met: metformin; yrs: years; IGT: Impaired glucose tolerance.

**Table 2:** Influence analysis assessment for the effect of lifestyle changes on T2D risk.

Author (Removed)	Risk Ratio (RR) with 95% CI	P-Effect	I^2^ (%)	P-Heterogeneity
Overall	0.53 [0.41, 0.67]	<0.001	63	0.01
Da Qing IGT And Diabetes Study (Pan, 1997 [32])	0.53 [0.41, 0.67]	<0.001	55	0.052
Diabetes Prevention Programme (Knowler, 2002 [34])	0.49 [0.37, 0.64]	<0.001	43	0.163
European Diabetes Prevention RCT—Newcastle (Penn, 2009 [40])	0.57 [0.44, 0.74]	<0.001	69	0.005
Finnish Diabetes Prevention Study (Tuomilehto, 2001 [33])	0.53 [0.41, 0.68]	<0.001	67	0.006
Indian Diabetes Prevention Programme (Ramachandran, 2006 [37])	0.54 [0.41, 0.72]	<0.001	57	0.038
Japanese Trial in IGT Males (Kosaka, 2005 [36])	0.48 [0.37, 0.63]	<0.001	67	0.006
Lifestyle Intervention on Metabolic Syndrome (Bo, 2007 [38,39])	0.54 [0.42, 0.69]	<0.001	66	0.008

CI = confidence interval.

**Table 3:** GRADE assessment for the effect of lifestyle changes on T2D risk.

Outcome	No. of Studies	Study Design	Certainty Assessment					RR [95% CI]	Certainty
			Risk of Bias	Inconsistency	Indirectness	Imprecision	Other Considerations		
T2D risk reduction	Seven	randomized trials	not serious	not serious ^a^	not serious	not serious	none	0.53 [0.41, 0.67]	⨁⨁⨁⨁ HIGH

CI = confidence interval; GRADE = grading of recommendations assessment, development, and evaluation; RR = risk ratio; T2D = type 2 diabetes. ^a^ Although there was significant heterogeneity (I^2^ = 65%, *p* = 0.01), the removal of one study [34] explained some of the heterogeneity, which changed it from significant to non-significant (I^2^ = 36%, *p* = 0.16). However, the estimate of effect did not change appreciably. Furthermore, this inconsistency was not considered serious as the magnitude of effect remained large and in the same direction across all the studies (RR < 0.72).

**Table 4:** Long-term post-intervention preventative effect on the incidence of type 2 diabetes in the former intervention groups compared to control groups in three randomized controlled lifestyle intervention studies.

Original Study	Risk Reduction	Comment
FDPS, Lindström J et al. Diabetologia 2013 [47]	Hazard Ratio 0.61, adjusted to 0.59 as compared to control group	Follow-up 13 years; follow-up data on the diet available
China Da Qing Diabetes Prevention Study, Li G et al. Lancet 2008 [46]	In total, 43% reduction in the combined intervention clinics as compared to control clinic	Follow-up 20 years; no detailed dietary data
Diabetes Prevention Program Group, Knowler WC et al. Lancet 2009 [49]	In total, 34% reduction in lifestyle intervention group and 18% reduction in metformin group as compared to placebo control group	Follow-up 10 year; no dietary data from the follow-up reported; long-term metformin use may modify the results

**Table 5:** Long-term post-intervention data on mortality, cardiovascular (CVD) mortality and microvascular complications in the former intervention groups compared to the control groups in three randomized controlled lifestyle intervention studies.

Original Study	Mortality	Cardiovascular Mortality	Reported Microvascular Complications
China Da Qing Diabetes Prevention Follow-up Study, Lancet Diabetes and Endocrinol, Gong Q et al., 2019 [54]	In total, 26% reduction in combined intervention clinics compared to original control group	In total, 33% reduction in combined intervention clinics compared to original control group	In total, 35% reduction in composite microvascular diseases and 40% reduction in any retinopathy in combined intervention clinics compared to original control group [54]
Diabetes Prevention Program Group, Lancet Diabetes and Endocrinol, Nathan DM et al., 2015 [55]	NA	NA	No group differences. Less microvascular complications in individuals who remained non-diabetic (RR 0.72, *p* < 0.001), less microvascular complications in intervention women (8.7% vs. control 11.0% or metformin groups, 11.2%, *p* = 0.03)
The Finnish Diabetes Prevention Follow-up Study PLoS One, Uusitupa M et al., 2009 [56] Nutrients, Aro A et al., 2019 [57]	NS between the original intervention and control groups	NS between the original intervention and control groups	Less early retinopathic changes in intervention (24% vs. 38%, adjusted odds ratio 0.52; 0.28–0.97, 95% CI, *p* = 0.039) than in control group; a subgroup analysis based on retinal photographs.

NA: Not available.
